# Impact of deuterated polyunsaturated fatty acids in neuroinflammation‐associated cognitive disfunction

**DOI:** 10.1002/alz70855_101647

**Published:** 2025-12-23

**Authors:** Beatriz Wagner, Mariana Chauvet, Sergio T. Ferreira, Alinny Isaac, Mychael V. Lourenco

**Affiliations:** ^1^ Federal University of Rio de Janeiro, Rio de Janeiro, Rio de Janeiro, Brazil; ^2^ Federal University of Rio de Janeiro, Rio de Janeiro, RJ, Brazil; ^3^ Federal University of Rio de Janeiro, Rio de Janeiro, Brazil; ^4^ Institute of Medical Biochemistry Leopoldo de Meis, Federal University of Rio de Janeiro, Rio De Janeiro, Rio de Janeiro, Brazil

## Abstract

**Background:**

Neuroinflammation, oxidative stress and lipid peroxidation comprise key events in neurodegenerative diseases, such as Alzheimer's disease, and are associated with neuronal damage, synaptic loss and cognitive decline. Recent studies have demonstrated that deuterated polyunsaturated fatty acids (D‐PUFA) are able to favor resolvin pathways (e.g. lipoxins) in detriment of the prostaglandin pathway, and therefore promote anti‐inflammatory effects. Our group has shown that lipoxin A4 protects against cognitive decline in animal models and is reduced in the liquor of patients with dementia. Here we investigate whether a diet enriched with D‐PUFAs is neuroprotective against neuroinflammation induced by LPS.

**Method:**

Three month‐old C57BL/6 male and female mice were fed with deuterated polyunsaturated fatty acid enriched diet (D‐PUFA) or control diet (H‐PUFA) for two months. Then, mice received intraperitoneal injections of LPS or vehicle. Behavior was tested by novel object recognition (NOR), displaced object recognition (DOR) and open field tasks.

**Result:**

All groups gained weight at the same rate during treatment and no gross difference in diet consumption was observed. Our results demonstrated that, whereas animals fed with control diet that received LPS presented deficits in NOR and DOR test, animals fed with D‐PUFA diet that received LPS did not present memory impairment. Brain tissues and plasma were collected for biochemistry analysis.

**Conclusion:**

Our findings suggest that D‐PUFA diet might be an interesting nutritional approach to mitigate neuroinflammation and cognitive decline.

